# The association between rs1260326 with the risk of NAFLD and the mediation effect of triglyceride on NAFLD in the elderly Chinese Han population

**DOI:** 10.18632/aging.203970

**Published:** 2022-03-25

**Authors:** Fan Yuan, Zhan Gu, Yan Bi, Ruixue Yuan, Weibo Niu, Decheng Ren, Lei Zhang, Guang He, Bao-Cheng Liu

**Affiliations:** 1 Bio-X Institutes, Key Laboratory for the Genetics of Developmental and Neuropsychiatric Disorders, Shanghai Jiao Tong University, Shanghai 200030, China; 2 Shanghai Innovation Center of TCM Health Service, Shanghai University of Traditional Chinese Medicine, Shanghai 201203, China

**Keywords:** NAFLD, GCKR, genetic association, triglyceride, mediation analysis

## Abstract

Background: Accumulated studies have pointed out the striking association between variants in or near *APOC3*, *GCKR*, *PNPLA3*, and nonalcoholic fatty liver disease (NAFLD) at various ages from multiple ethnic groups. This association remained unclear in the Chinese Han elderly population, and whether this relationship correlated to any clinical parameters was also unclear.

Objectives: This study aims to decipher the complex relevance between gene polymorphisms, clinical parameters, and NAFLD by association study and mediation analysis.

Methods: Eight SNPs (rs2854116, rs2854117, rs780093, rs780094, rs1260362, rs738409, rs2294918, and rs2281135) within *APOC3*, *GCKR,* and *PNPLA3* were genotyped using the MassARRAY® platform in a large Chinese Han sample comprising of 733 elderly NAFLD patients and 824 age- and ethnic-matched controls. Association and mediation analysis were employed by R.

Results: The genotypic frequencies of rs1260326 and rs780094 were significantly different between NAFLD and control (rs1260326: *P*=0.004, *P_corr_*=0.020, OR [95%CI]= 0.69 [0.54-0.89]; rs780094: *P*=0.005, *P_corr_*=0.025, OR [95%CI]= 0.70 [0.55-0.90]). Particularly, an increased triglyceride level was observed in carriers of rs1260326 T allele (1.94±1.19 mmol/L) compared with non-carriers (1.73±1.05 mmol/L).no significant results were observed in rs780094. Notably, triglyceride levels had considerably indirect impacts on association between NAFLD and rs1260326 (β =0.01, 95% CI: 0.01–0.02), indicating that 12.7% of the association of NAFLD with rs1260326 was mediated by triglyceride levels.

Conclusions: Our results identified a prominent relationship between *GCKR* rs1260326 and NAFLD, and highlighted the mediated effect of triglyceride levels on the that association in the Chinese Han elderly.

## INTRODUCTION

Nonalcoholic fatty liver disease (NAFLD) is defined by lipid deposition exceeding more than 5% of hepatocytes and/or more than 5.6% hepatocellular fat content per weight unit of liver without significant alcohol consumption and other causes of fatty liver [1, 2]. It has been the most prominent cause of chronic liver disease worldwide with the global prevalence being around 25% [3].

NAFLD is considered to possess a complex trait resulting from environmental exposures and multiple susceptible genes. According to available data, the heritability was estimated to range from 20% to 70% [4]. The exact pathogenesis of NAFLD is not clarified completely, but increasing evidence supported the role of single nucleotide polymorphism (SNP) in the risk and development of NAFLD, especially SNPs within those genes associated with lipid handling and oxidative stress, such as the patatin-like phospholipase domain containing protein 3 (*PNPLA3*), glucokinase regulatory protein gene (*GCKR*), and apolipoprotein C3 (*APOC3*) gene [5, 6].

*GCKR* was previously well-described to be involved in the development of NAFLD in children and adolescents [7]. Some genome-wide association studied (GWAS) and meta-analyses showed that GCKR rs780094, rs780094, and rs1260326 were closely related to the risk of NAFLD in Japanese [8], Iran [9], Danish [10], and Swedish [11] populations. In China, it is reported that *GCKR* polymorphism was associated with NAFLD in the Uyghur population [12]. However, in the Han population, especially in the elderly population, more studies are necessary to be carried out. Notably, the researchers found that rs780093 was associated with triglyceride (TG) levels in Europeans, which is a risk factor for NAFLD [13].

Meanwhile, *PNPLA3* SNPs were also reported to be relevant to lower NAFLD risk in a population comprising Hispanic, African American, and European American individuals [14]. *PNPLA3* rs738409 and rs2294918 influence the hepatic fat content in India by an exome-wide approach [15, 16]. Another *PNPLA3* SNPs, rs2281135, showed a significant association with the NAFLD in a Korean [17].

To our interest, the relationship between NAFLD and *APOC3* promoter region SNPs rs2854117 and rs2854116 is controversial in different studies. It has been proposed that rs2854116 and rs2854117 were associated with NAFLD in lean individuals of South Asian descent [18]. On the contrary, Federica et al. did not identify any significant association between these two *APOC3* SNPs and NAFLD in Southern Europeans [19].

Overall, the association between *APOC3*, *GCKR*, *PNPLA3* gene and NAFLD in the elderly Chinese Han population remains unclear or controversial. We carried on our study to explore the relationship between several SNPs within these three genes, NAFLD and clinical parameters in the elderly Han Chinese population to decipher the complex relationship between gene polymorphisms, clinical parameters, and the risk of NAFLD.

## MATERIALS AND METHODS

### Subjects

This study was conducted during 2015 and 2016 in Shanghai, China. Initially, 765 NAFLD patients and 860 ethnic- and age-matched healthy controls were recruited in this study. NAFLD was defined by evidence of hepatic steatosis on B-mode Philips ClearVue 550 ultrasound system with a 3.5MHz C5-1 broadband curved array transducer (Philips Medical System, Bothell, WA, USA), and evaluated by two expert and board-certified radiologists. NAFLD was diagnosed according to the guidelines for managing NAFLD of the Chinese Medical Association in 2010 [20]. All the subjects should meet the following standards: 1) all above the age of 60; 2) permanent residents of the Zhangjiang area in Pudong district, Shanghai.;3) no alcohol abuse (< 140g/week for male and < 70g/week for female); 4) Chinese Han population with no blood relation to each other; 5) free of drug-induced liver disease or autoimmune liver disease; 6) no carriers of hepatitis B or C. The Ethics Committee in Shanghai University of Traditional Chinese Medicine approved this study. Informed consents were obtained from all subjects.

### Clinical parameters

The baseline information such as age, gender, alcohol consumption, current smoking, and medical history were collected by questionnaire. The height and body weight were measured by an electronic measurement instrument (Shengyuan, Zhengzhou, China). Body mass index (BMI) was calculated according to the formula body weight/height^2^ (kg/m^2^). Systolic Blood Pressure (SBP) and Diastolic Blood Pressure (DBP) were measured by electronic sphygmomanometers (Biospace, Cheonan, South Korea).

Blood samples were obtained from subjects after an overnight fasting period. Fasting plasma glucose (FPG), total cholesterol (TC), TG, low-density lipoprotein cholesterol (LDL-C), high-density lipoprotein cholesterol (HDL-C), alanine transaminase (ALT), alkaline phosphatase (ALP), aspartate transaminase (AST), and gamma-glutamyl transferase (GGT) were measured using an automatic biochemistry analyzer (Hitachi, Tokyo, Japan).

### Genotyping

Genomic DNA was extracted from venous blood leukocytes by the standard phenol-chloroform method. Totally, eight SNPs were selected from the literature and the National Center for Biotechnology Information dbSNP database (http://www.ncbi.nlm.nih.gov/SNP) for Genotyping by a matrix-assisted laser desorption/ionization time-of-flight (MALDI-TOF) mass spectrometer using the MassARRAY^®^ Analyzer 4 platform (Sequenom, CA, USA). The SNPs information included in the final analysis is listed in Table 1. The minor allele frequency (MAF) of SNPs in our present study was comparable to that in East Asian population reported in the 1000 Genomes Project [21]. In order to ensure the reliability of genotyping quality, quality control was carried out at both an individual level and a SNP level [22]. At the individual level, subjects with incomplete information were excluded. In addition, individuals with a call rate of less than 0.8 were also excluded. SNPs that violated Hardy-Weinberg equilibrium (HWE<0.05) were removed at the SNP level. Moreover, no template controls (>1%) were called blind to their status in the genotyping process and each SNP was re-genotyped in at least 5% random DNA samples.

**Table 1:** The SNPs analyzed in this study.

**SNP**	**Gene**	**Location**	**Alleles**	**Chromosome position^1^**	**MAF_G**	**MAF_E**	**MAF_P**
rs2854116	*APOC3*	Upstream	C>T	11:116829453	T=0.452	C=0.470	C=0.435
rs2854117	*APOC3*	Upstream	T>A / T>C	11:116829426	C=0.499	T=0.474	T=0.432
rs1260326	*GCKR*	Missense	T>C / T>G	2:27508073	C=0.293	C=0.481	C=0.453
rs780094	*GCKR*	Intron	T>C	2:27518370	C=0.302	C=0.476	C=0.462
rs780093	*GCKR*	Intron	T>C	2:27519736	C=0.292	C=0.480	C=0.467
rs738409	*PNPLA3*	Missense	C>G / C>T	22:43928847	G=0.262	G=0.350	G=0.347
rs2294918	*PNPLA3*	Missense	A>G	22:43946236	A=0.212	A=0.192	A=0.195
rs2281135	*PNPLA3*	Intron	G>A / G>C	22:43936690	A=0.257	A=0.364	A=0.354

^1^The SNP Chromosome positions are based on the NCBI human genome build GRCh38.p12.

MAF_G, minor allele frequency in global; MAF_E, minor allele frequency in East Asian; MAF_P, minor allele frequency in present study.

Global and East Asian allele frequencies information was collected from 1000 genomes project [21].

### Statistical analysis

Continuous variables like age and BMI were presented as the mean ± standard error and analyzed by t-test. Categorical variables like gender were describe as a percentage and analyzed by chi-square test. The HWE, allelic and genotypic distribution was examined using an R package called SNPassoc (https://cran.r-project.org). For pairwise linkage disequilibrium (LD) analysis, Haploview 4.2 (Broad Institute, Cambridge, MA, USA) was carried out. To analyze the eight SNPs, we perform Bonferroni correction (*P_corr_* =*P*-value*8) to prevent inflation of the type I error. Mediation models were established to explore whether TG mediated the association between SNP and NAFLD by an R-package called mediation. *P*-values were two tailed and the threshold of statistical difference was set at *P_corr_* <0.05. Furthermore, the illustration was created with BioRender.com (https://biorender.com).

## RESULTS

### Demographics of study subjects

1625 subjects were initially enrolled in this study. 51 patients were removed due to the absence of blood samples, personal information, or failure of genotyping. 1557 individuals were in the final analysis consisting of 733 NAFLD patients and 824 healthy controls. Details about age, gender, BMI, and clinical characteristics (Diabetes, Hypertension, Hypertriglyceridemia, Hypercholesteremia and Hyperuricemia) among NAFLD patients and control were shown in Table 2. There were no significant differences in age, gender, the percentage of hypercholesteremia and hyperuricemia between NAFLD and control groups. Nevertheless, BMI was significantly higher in NAFLD (NAFLD: 26.11 ± 3.269 kg/m^2^ VS. Control: 24.28 ± 3.474 kg/m^2^; *P* < 0.05). The percentages of Diabetes (NAFLD: 25.10% VS. Control: 20.63%; *P* = 0.036), hypertension (NAFLD: 63.44% VS. Control: 51.21%; *P* = <0.001), and hypertriglyceridemia (NAFLD: 55.14% VS. Control: 40.86%; *P* = <0.001) were also remarkably higher in NAFLD.

**Table 2:** Demographic and clinical characteristics of subjects.

	**Control (824)**	**NAFLD (733)**	***P***
Age (years)	71.66 ± 6.394	71.47 ± 6.682	0.560
BMI (kg/m^2^)	24.28 ± 3.474	26.11 ± 3.269	<0.001*
Gender (male, %)	42.48	40.38	0.403
Diabetes (%)	20.63	25.10	0.036*
Hypertension (%)	51.21	63.44	<0.001*
Hypertriglyceridemia (%)	40.86	55.14	<0.001*
Hypercholesteremia (%)	26.44	25.68	0.773
Hyperuricemia (%)	16.23	17.57	0.521

BMI, body mass index; *statistically significant.

### Genetic association between SNPs and NAFLD

As is described in Table 3, the allelic distributions of all SNPs were in HWE (all *P*>0.05). The frequency of C allele of *GCKR* rs780094 was significantly lower in NAFLD than in control (OR= 0.867, 95%CI= 0.75-0.99; *P*= 0.048). However, this result did not survive after Bonferroni correction (*P_corr_*= 0.384).

**Table 3:** Allelic distribution of SNPs between NAFLD and control.

**SNP**		**T/C**	**MAF**	**HWE**	**chi2**	**OR [95% CI]**	***P***	***P_corr_***
rs2854116	NAFLD	826/640	0.436	0.69	0.023	1.011	0.879	>0.999
	control	933/715	0.433			[0.87-1.16]		
		C/T						
rs2854117	NAFLD	825/641	0.437	0.088	0.319	1.041	0.571	>0.999
	control	944/704	0.427			[0.90-1.20]		
		T/C						
rs1260326	NAFLD	829/637	0.434	0.759	3.735	0.869	0.053	0.424
	control	875/773	0.469			[0.75~1.00]		
		T/C						
rs780094	NAFLD	816/650	0.443	0.541	3.906	0.867	0.048*	0.384
	control	859/789	0.478			[0.75-0.99]		
		T/C						
rs780093	NAFLD	808/659	0.448	0.541	3.639	0.871	0.056	0.448
	control	852/796	0.485			[0.75-1.00]		
		C/G						
rs738409	NAFLD	952/514	0.35	0.655	0.175	1.032	0.674	>0.999
	control	1082/566	0.343			[0.89-1.19]		
		G/A						
rs2294918	NAFLD	1191/271	0.185	0.145	1.605	0.891	0.205	>0.999
	control	1308/334	0.203			[0.74-1.06]		
		G/A						
rs2281135	NAFLD	943/523	0.356	0.868	0.099	1.023	0.752	>0.999
	control	1069/579	0.351			[0.88-1.18]		

MAF, minor allele frequency; HWE, Hardy-Weinberg equilibrium; OR, odds ratio; CI, confidence interval; *P*_corr_, *P* value after Bonferroni correction; *statistically significant.

**Figure 1:**
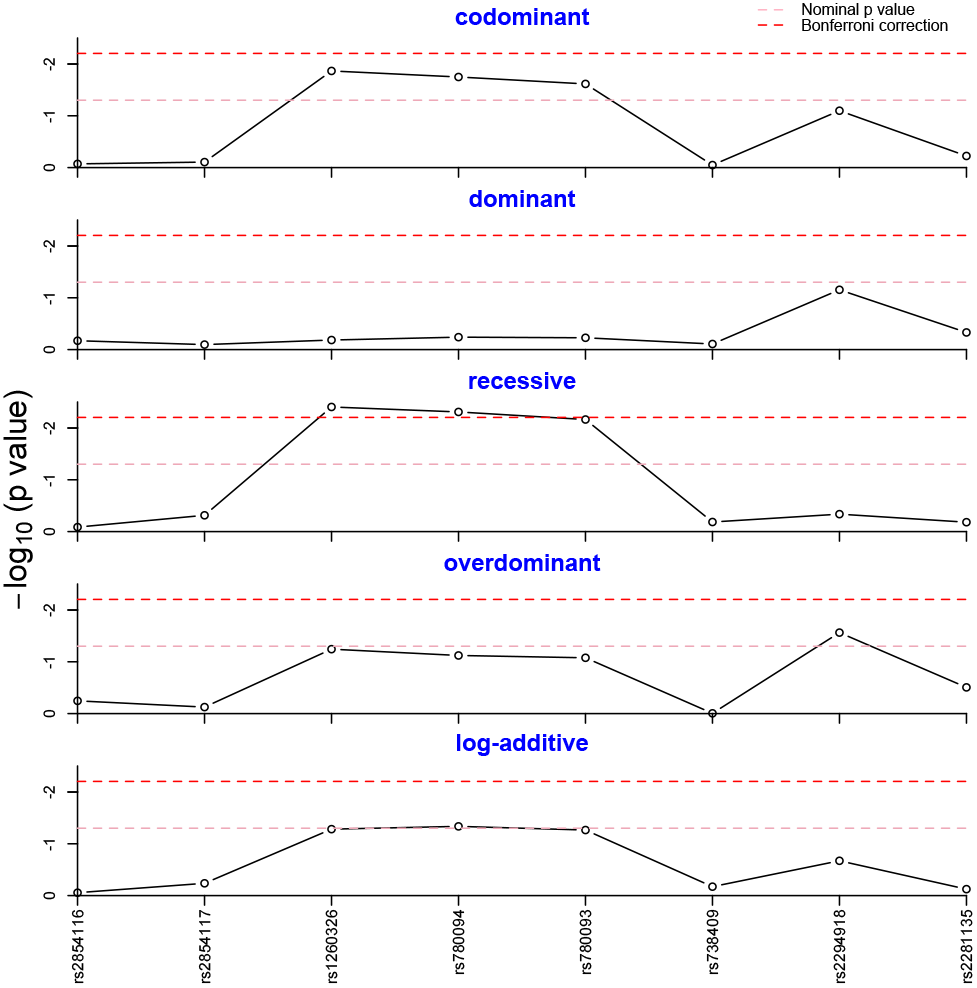
**Genotype frequency analysis in five genetic models.** The genotype frequencies of rs1260326 and rs780094 were statistically different in the recessive model after Bonferroni correction.

Five different genetic models (Codominant, Dominant, Recessive, Overdominant, and Log-additive) of each SNP were tested to assess their association with NAFLD further. As shown in Figure 1, the recessive model of rs1260326 was still statistically significant after Bonferroni correction. Likewise, the significant result of rs780094 survived in recessive model after Bonferroni correction. No other SNPs obtained significant differences in any genetic model.

### Association of *GCKR* rs1260326 variant with NAFLD and clinical parameters

The detailed genotypic distributions of rs1260326 are listed in Table 4. A significantly different result was observed in the recessive model after Bonferroni correction. The rs1260326 CC genotype was remarkably related to decreased risk of NAFLD (OR=0.69; 95%CI=0.54-0.89; *P*_corr_ =0.020). In addition, the association remained significant after adjusting for age, gender and BMI (OR=0.70; 95%CI=0.54-0.91; *P*_corr_ =0.035). These results suggested that *GCKR* rs1260326 polymorphism is associated with the risk of NAFLD in Chinese Han elderly population.

**Table 4:** *GCKR* rs1260326 genotype distributions in five genetic models.

		**Control (%)**	**NAFLD (%)**	**OR**	**95% CI**	***P***	***P_corr_***	**OR^1^**	**95% CI^1^**	***P* ^1^**	***P_corr_* ^1^**
Codominant	T/T	29.2	30.3	1		0.013*	0.068	1		0.247	>0.999
	T/C	47.7	52.5	1.06	[0.84-1.34]			0.98	[0.77-1.24]		
	C/C	23.1	17.2	0.72	[0.54-0.96]			0.69	[0.51-0.93]		
Dominant	T/T	29.2	30.3	1		0.654	>0.999	1		0.291	>0.999
	T/C+C/C	70.8	69.7	0.95	[0.77-1.18]			0.89	[0.71-1.11]		
Recessive	T/T+T/C	76.9	82.8	1		0.003*	0.020*	1		0.007*	0.035*
	C/C	23.1	17.2	0.69	[0.54-0.89]			0.7	[0.54-0.91]		
Overdominant	T/T+C/C	52.3	47.5	1		0.057	0.285	1		0.223	>0.999
	T/C	47.7	52.5	1.21	[0.99-1.48]			1.14	[0.92-1.40]		
log-Additive	0,1,2	52.9	47.1	0.87	[0.75-1.00]	0.052	0.261	0.84	[0.73-0.98]	0.025*	0.125

In additive genetic model, genotypes were coded as 0, 1, or 2 according to the number of minor allele for a specific individual. *P* value after Bonferroni correction; *statistically significant.

^1^Associations were tested using logistic regression with adjustment for age, gender, and BMI.

**Figure 2:**
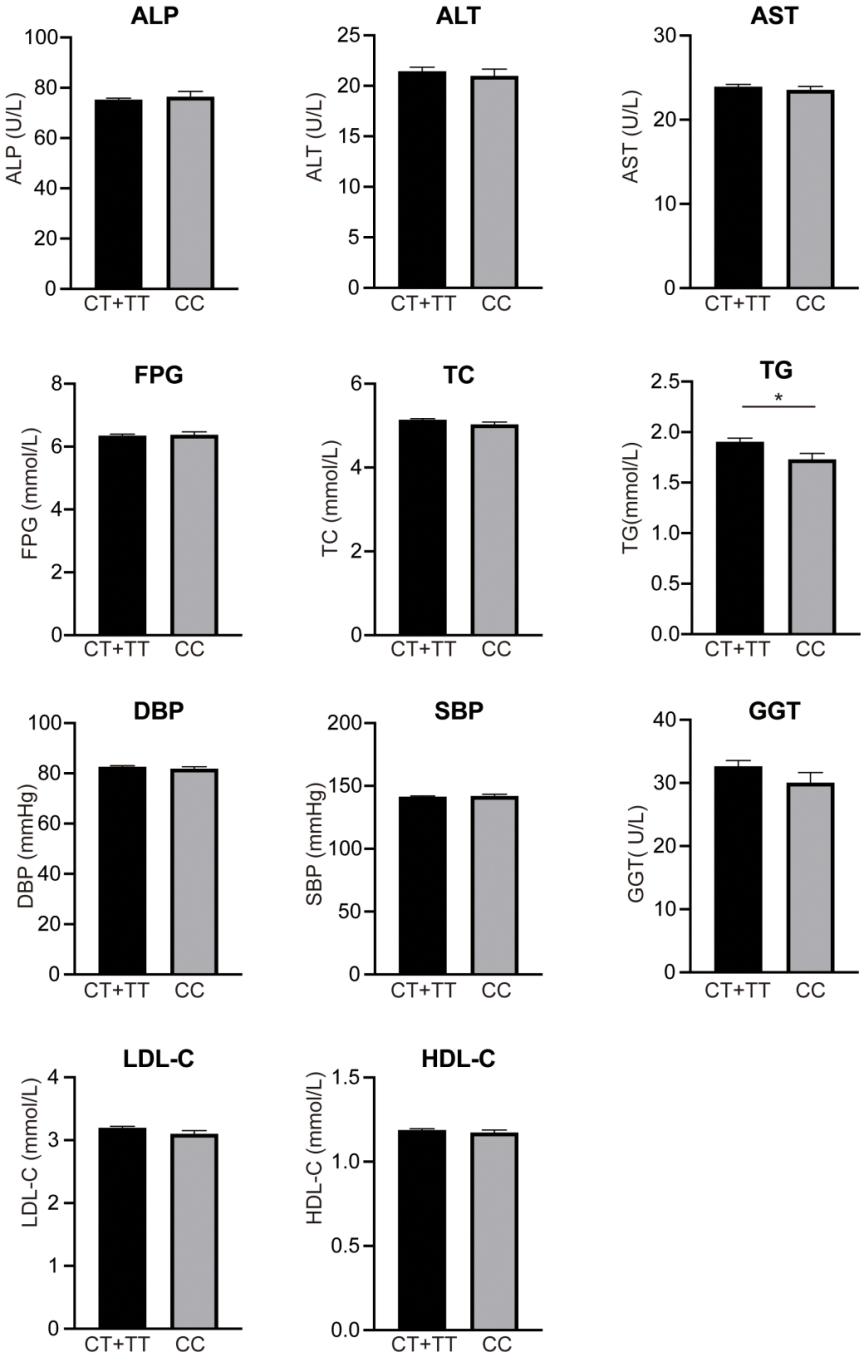
**Levels of clinical parameters between rs1260326 CC and genotypes.** TG level in carriers of rs1260326 T allele (1.94±1.19 mmol/L) was significantly higher than non-carriers (1.73±1.05 mmol/L). However, no marked difference of other clinical parameters was observed between the carriers and non-carriers of rs1260326 T allele (*p* > 0.05). ALP, alanine Phosphatase; ALT, alanine transaminase; AST, aspartate transaminase; FPG, fasting plasma glucose; TC, total cholesterol; TG, triglyceride; SBP, systolic blood pressure; DBP, diastolic blood pressure; GGT, glutamyl transpeptidase; LDL-C, low-density lipoprotein cholesterol; HDL-C, high-density lipoprotein cholesterol; * *p* < 0.05. All data are represented as mean ± s.e.m.

The associations between the *GCKR* rs1260326 variant and the hepatic enzyme, lipid, blood pressure, and FBG levels are shown in Figure 2. An increased TG level was observed in carriers of rs1260326 T allele (1.94±1.19 mmol/L) compared with non-carriers (1.73±1.05 mmol/L). However, no marked difference of other clinical parameters was observed between the carriers and non-carriers of the rs1260326 T allele (*p* > 0.05).

### Association of *GCKR* rs780094 variant with NAFLD and clinical parameters

The detailed genotypic distributions of rs780094 were listed in Table 5. A significantly different result was observed in the recessive model after Bonferroni correction. rs780094 CC genotype was remarkably related to decreased risk of NAFLD (OR=0.70; 95%CI=0.55-0.90; *P*_corr_ =0.025). In addition, the association remained significant safter adjusting for age, sex and BMI (OR=0.70; 95%CI=0.54-0.90; *P*_corr_ =0.030). However, we did not find any differences in clinical parameters between rs780094 CC and CT+TT genotype (data is not shown here).

**Table 5:** *GCKR* rs780094 genotype distributions in five genetic models.

		**Control (%)**	**NAFLD (%)**	**OR**	**95% CI**	***P***	***P_corr_***	**OR^1^**	**95% CI^1^**	***P* ^1^**	***P_corr_* ^1^**
Codominant	T/T	27.9	29.2	1		0.018	0.09	1		0.022*	>0.999
	T/C	48.4	52.9	1.05	[0.83-1.32]			0.97	[0.77-1.24]		
	C/C	23.7	17.9	0.72	[0.54-0.96]			0.69	[0.51-0.93]		
Dominant	T/T	27.9	29.2	1		0.576	>0.999	1		0.273	>0.999
	T/C+C/C	72.1	70.8	0.94	[0.75-1.17]			0.88	[0.71-1.11]		
Recessive	T/T+T/C	76.3	82.1	1		0.005	0.025*	1		0.006*	0.030*
	C/C	23.7	17.9	0.7	[0.55-0.90]			0.7	[0.54-0.90]		
Overdominant	T/T+C/C	52.9	47.1	1		0.076	0.38	1		0.211	>0.999
	T/C	48.4	52.9	1.2	[0.98-1.46]			1.14	[0.54-0.90]		
log-Additive	0,1,2	52.9	47.1	0.86	[0.75-1.00]	0.046	0.23	0.84	[0.72-0.97]	0.021*	>0.999

In additive genetic model, genotypes were coded as 0, 1, or 2 according to the number of minor allele for a specific individual. *P* value after Bonferroni correction; *statistically significant.

^1^Associations were tested using logistic regression with adjustment for age, gender, and BMI.

### Mediated effect of TG on the association of rs1260326 and NAFLD

**Figure 3:**
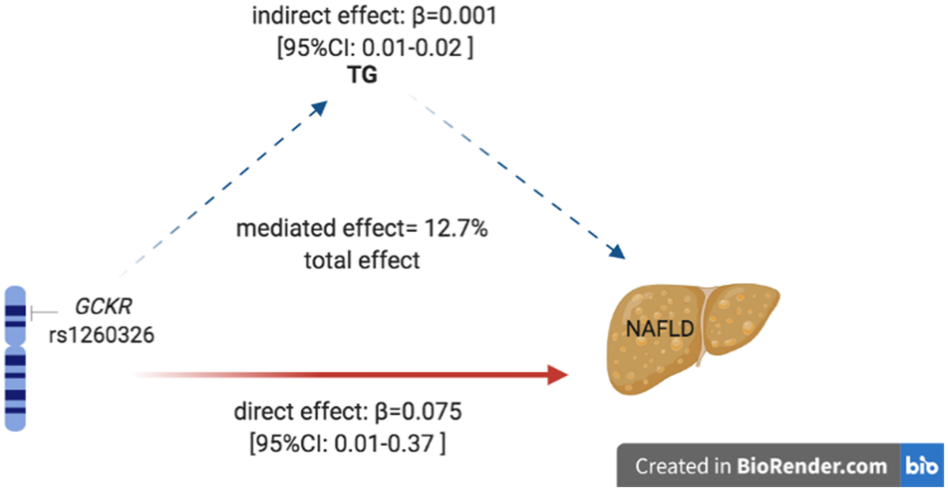
**Mediation of TG on the association between rs1260326 and NAFLD.** Zero was not included in 95% confidence intervals representing statistical significance. TG, triglyceride; NAFLD, non-alcoholic fatty liver disease.

As reported above, the rs1260326 polymorphism was associated with TG level and NAFLD risk. Also, we found that the TG level was correlated with NAFLD, suggesting that the mechanism underlines the association between rs1260326 and NAFLD was possibly mediated by TG level. We conducted mediation analysis to explore whether TG mediated the association between rs1260326 and NAFLD. As shown in Figure 3, mediation analysis indicated that rs1260326 had a significant direct effect on NAFLD incidence (β =0.74, 95% CI: 0.02–0.13, P<0.001), and TG mediated the indirect effect on NAFLD incidence by 12.7% (β =0.01, 95% CI: 0.01–0.02).

### Haplotype analysis

**Figure 4:**
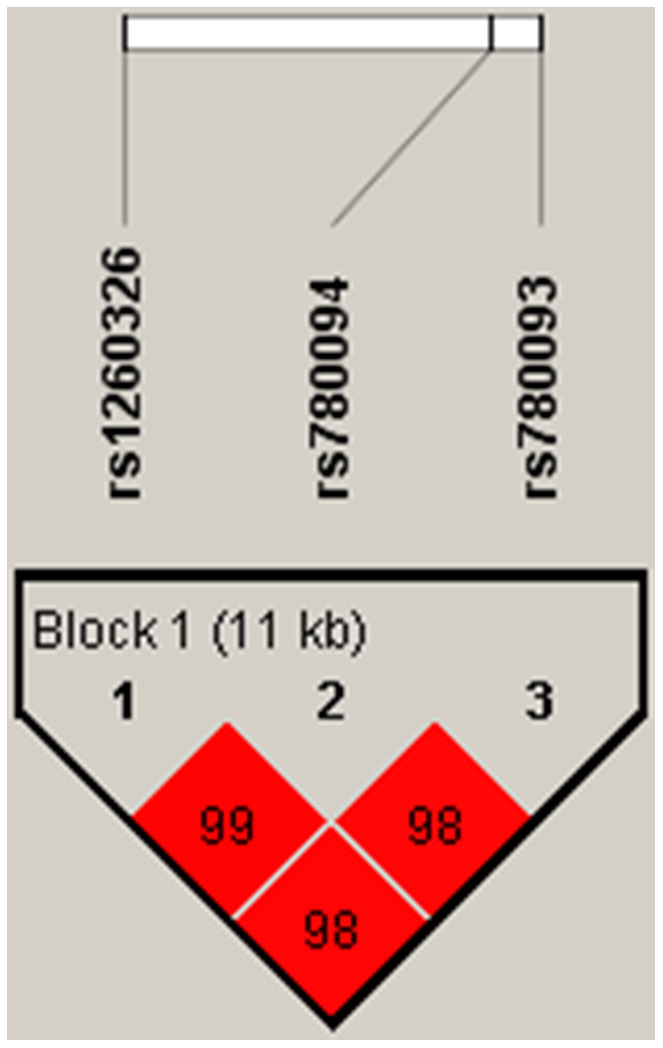
**Linkage disequilibrium plot between SNPs.** The number in each square is r^2^*100 between two SNPs. As shown in the picture above rs1260326-rs780094-rs780093 was identified as a strong block with r^2^>0.8.

We identified rs1260326-rs780094-rs780093 as a strong LD block in the *GCKR* gene with Haploview analysis (Figure 4).

## DISCUSSION

To the best of our knowledge, this is the first time investigating the relationship between *APOC3*, *GCKR*, *PNPLA3* gene polymorphisms with NAFLD and clinical parameters in the Chinese Han elderly. Here, we analyzed two *APOC3* SNPs (rs2854116 and rs2854117)*,* three *GCKR* SNPs (rs780093, rs780094, and rs1260326), and three *PNPLA3* SNPs (rs738409, rs2294918, and rs2281135) in 1557 Chinese Han elderly subjects. We found that rs780094 and rs1260326 were significantly associated with NAFLD in the elderly Chinese Han population. Of note, rs1260326 T allele was related to higher TG levels, and about 12.7% of the rs1260326 effect on NAFLD was mediated through TG levels.

The glucokinase regulatory protein, translated by GCKR gene, is an inhibitor of glucokinase (GCK) activity which is the principal hexokinase in the liver. GCK functioned as a glucose sensor to regulate glucose metabolism and has been reported to be closely related to hepatic insulin sensitivity, playing a vital role in the development of NAFLD [23–25]. Moreover, *GCKR*-deficient mice supported that the disruption of *GCKR* regulation could cause glycemic control impairment [26]. N. Santoro et al. found that *GCKR* gene variant was associated with NAFLD in children and adolescents [27]. However, few studies were focusing on the elderly. The rs780094 SNP within *GCKR* gene was associated with liver fat accumulation, increased triglyceride concentrations, reduced insulin levels, and reduced risk of type 2 diabetes [28, 29]. Inconsistent results have been reported about the effects of *GCKR* polymorphisms on the risk of NAFLD, probably due to the ethnic differences among the NAFLD patients studied [30–32]. We confirmed the result reported by Yang et al. with a larger sample size and older population [32].

Nonsynonymous rs1260326 SNP (C/T, P446L substitution) was identified as a strong signal for total triglycerides concentrations [33]. A study on Caucasian, American, and Iceland populations indicated that variant in rs1260326 may cause GCKR inhibitory function to defect, leading to increased glucokinase activity and hepatic glucose uptake [7]. Additionally, the rs780094 polymorphism was related to elevated type 2 diabetes risk, which may indirectly influence the risk of developing NAFLD [34]. Triglyceride levels had considerably indirect impacts on association between NAFLD and rs1260326.

Our study is the first one to investigate the modulation of the association between rs1260326 and NAFLD in the elderly Chinese Han population by TG concentration. A recent work genotyped five GCKR SNPs and found they were associated with increased TG levels, in which rs1260326 was included [35]. Several molecular mechanisms can explain our findings - they were based on increased glucose uptake associated with GCKR SNP. Firstly, the *GCKR* rs1260326 T allele was associated with increased TG concentration. Furthermore, a meta-analysis in the European population confirmed this association between rs1260326 T allele and higher serum TG level [36]. Consistently, our present study also revealed the higher TG concentration in carriers of the rs1260326 T allele. In a cross-sectional study, TG/HDL-C was independently related to NAFLD [37]. The mediation statistical model enables researchers to infer why or how the two variables are related, rather than just determining whether the results occur. NAFLD is considered to possess a complex trait resulting from environmental exposures and multiple susceptible genes. Our study is the first research to explore the correlation and causal mediation between TG level and NAFLD in Chinese elderly group even though the mediation effect in our study is not as high as that in Nichols, P. H. et al. result - the mediated effect of TSH on NAFLD was 16.0% [38]. Our mediation analysis showed that TG played a partial mediating role in the relationship between rs1260326 and NAFLD. Our findings provide evidence for the mechanistic role of increased TG levels in the association between rs1260326 and NAFLD.

In our study, the diagnosis method of NAFLD is an alternative to histological diagnosis, as the latter is difficult to obtain and invasiveness. The last but not least, a meta-analysis proved that ultrasound is a reliable and accurate method for detecting moderate to severe fatty liver disease, with non-invasive, low cost, high safety, and good availability [39]. In general, despite the limitations of insufficient SNP coverage in candidate genes and not generalized to the entire population, further genetic studies are needed to evaluate further or confirm the role of *GCKR* gene in NAFLD.

## CONCLUSIONS

In summary, our results demonstrated that the *GCKR* SNPs rs780094 and rs1260326 might be associated with NAFLD in the elderly Chinese Han. Besides, the rs1260326 T allele was related to higher TG concentration. Of note, TG concentration has partly an indirect effect on the observed association between the *GCKR* rs1260326 SNP and NAFLD. Our finding provided a reference for future studies on the *GCKR* in predicting the NAFLD risk.
